# Enhanced nutrient and organic matter removal from dairy wastewater through an optimized activated algae process

**DOI:** 10.3389/fpls.2026.1721052

**Published:** 2026-03-23

**Authors:** Olga Tiron, Luoana Florentina Pascu, Tatiana Buse, Valeriu Robert Badescu, Laurentiu Razvan Dinu, Mirela Alina Constantin, Lucian Alexandru Constantin

**Affiliations:** National Research and Development Institute for Industrial Ecology - ECOIND, Bucharest, Romania

**Keywords:** activated algae, dairy wastewater, microalgae harvesting, microalgae-bacteria consortium, photosynthetic oxygen, wastewater treatment

## Abstract

Wastewater treatment faces increasing pressure to transition from energy-intensive technology to sustainable alternatives aligned with global resource efficiency and climate goals. Microalgae-based processes have emerged as promising solutions in environmental remediation applications; however, their large-scale deployment remains constrained by contamination risks, stringent operational requirements, and high downstream costs. These challenges are particularly evident in the treatment of nutrient-rich industrial influents such as dairy wastewater, which represents an environmental concern. Addressing this gap is important for strengthening overall climate action efforts and safeguarding ecosystems by reducing greenhouse gas emissions and transforming nutrient loads from pollution sources into potential resources. In this study, a two-stage biological treatment of raw dairy wastewater was tested as an alternative to conventional technology. The process relied on activated algae biomass, consisting of microalgae-bacteria consortia, operated in sequencing batch mode. The treatment stages were strategically designed to address elevated organic and ammonium loads while maintaining aerobic conditions exclusively through photosynthesis. The first stage operated at high COD loadings (>1 g O_2_L) and achieved organic matter removal above 80%, while the second stage, adapted to lower COD (<0.5 g O_2_L), ensured residual ammonium below the detection limit and overall COD removal up to 99%. Optimization of operational conditions further improved microalgae harvesting efficiency (from 88.6 ± 2.7% to 94.4 ± 1.8%) and enhanced floc stability through diversification of microalgae communities. Complementary, microfauna analysis outlined the presence of protozoan and metazoan populations confirming process stability and ecological balance comparable to traditional activated sludge system. The findings demonstrate potential of the activated algae system as a resilient and resource-efficient alternative to conventional wastewater treatment technology. By avoiding energy demand for mechanical aeration and ensuring nutrient recovery in line with environmental regulatory frameworks, the developed process supports sustainable wastewater treatment management while aligning with international goals on climate change mitigation and aquatic ecosystems protection.

## 1 Introduction

The demand for water resources required in various anthropogenic activities is increasing simultaneously with the development of socio-economic systems. Unfortunately, available freshwater resources are limited and cannot be substituted by any other alternative sources (European Commission, 2012). Considering the sustainable development strategic approaches, protecting and conserving water resources has been consistently highlighted as a priority target, emphasized in the 2030 Agenda for Sustainable Development (United Nations, 2015) and reinforced continuously by frameworks and assessments linking water management with sustainability and bioeconomy policies (UN-Water, 2023; United Nations Environment Programme, 2022; Warchold and Pradhan, 2025). Wastewater treatment plays a central role in these efforts, as effluents must comply with regulatory limits before discharge to ensure safe reintroduction into the hydrological cycle. However, traditional wastewater treatment, based on the activated sludge process, encounters important economic and environmental challenges. These include greenhouse gas emissions (Bao et al., 2016), high energy consumption during the aerobic treatment stage (accounting for 45-75% of total operating costs; Stenstrom and Rosso, 2008; Ozturk et al., 2016; Tao et al., 2024), and sludge management concerns (Cieślik et al., 2015; Vaithyanathan and Cabana, 2021). Therefore, conventional treatment technology is a source of pollution, resource-intensive, and costly, which further limits its scalability, particularly in resource limited areas. Globally, only about half of all collected wastewater is treated, with significant regional differences, from less than 4% in low-income to more than 75% in high-income countries (UN-Water, 2023). To achieve target 6.3 of the United Nations Sustainable Development Goals (halving the proportion of untreated wastewater and increasing recycling and safe reuse globally by 2030), international reports emphasize the urgent need for more sustainable approaches, including nature-based solutions capable of delivering treatment efficiency while also providing co-benefits for ecosystems and communities.

To address these limitations, microalgae-based systems have been explored as a beneficial alternative (Batista et al., 2015; Acién et al., 2018; Osuna-Martínez et al., 2025; Tianqi et al., 2025). Such an approach can contribute to wastewater treatment plants (WWTPs) and utility operators by: (*i*) reducing costs for oxygen supply by leveraging oxygen produced via photosynthesis by photoautotrophic microalgae, (*ii*) mitigating CO_2_ emissions during aerobic treatment through biofixation in microalgae cells, and (*iii*) converting biomass waste into value-added products, aligning with the increasing interest in using microalgae biomass as a raw material for a wide range of applications (Daud et al., 2015; Praveen and Loh, 2015; Molazadeh et al., 2019; Ampofo and Abbey, 2022; Srimongkol et al., 2022; Ramírez Mérida and Rodríguez Padrón, 2023). The average turnover generated by enterprises dealing with microalgae during 2016–2021 in European countries was estimated at around 31.6 million euros (Vásquez and Sanchez, 2022).

However, microalgae culturing requires high costs related to upstream (biomass cultivation) and downstream processing steps, including harvesting and dewatering (Bhatt et al., 2022). One proposed strategy to decrease cultivation costs is the use of wastewater as a culture medium to provide necessary nutrients (Acién et al., 2012; Prandini et al., 2016; Rafa et al., 2021). This strategy has been reinforced by recent European initiatives, which highlight the role of algae in nutrient recycling, carbon capture, water quality improvement and alignment with circular bioeconomy strategies supporting both the Green Deal objectives and the Sustainable Development Goals (European Commission, 2025a; 2025b). As a result, there is an increasing interest in applying microalgae-based technology for the treatment of different types of wastewater, including municipal, tannery, agricultural, pharmaceutical, and petrochemical effluents (Abinandan and Shanthakumar, 2015; Gallego et al., 2025). Furthermore, valorization of resulting biomass may reduce costs or generate revenue, helping offset upstream expenditures (Morais et al., 2023).

Dairy wastewater is characterized by high organic matter and nutrient loading and represents one of the largest volumes of industrial effluents generated within the food processing sector (Usai et al., 2024). In the EU-27, the annual volume of dairy wastewater was estimated at 192.5 million m³ for 2018, with nearly half originating from cheese production (Stasinakis et al., 2022). On a global scale, for each liter of milk processed, between 1 and 6 liters of wastewater are generated, with some assessments reporting values up to 10 L depending on product type and cleaning practices (Tabelini et al., 2023; Singh et al., 2024). Due to these characteristics, conventional biological treatment of dairy wastewater based on the activated sludge process is highly energy-demanding, mainly because of intensive aeration required to sustain the aerobic stage, involves extended hydraulic retention times (HRTs) of up to 8 days, and leads to large amounts of waste sludge with limited valorization options (Kushwaha et al., 2011; Yu et al., 2023). The generation of dairy processing sludge in the EU was estimated at approximately 2.45 million tons (wet weight) for 2020, further highlighting the environmental burden of this sector (Hu et al., 2021).

Microalgae–bacteria biomass has been investigated as an alternative for organic matter and nutrient removal from dairy wastewater, with several drawbacks identified. Reported limitations include an imbalance between COD and ammonium removal efficiencies, high HRT requirements, and poor settling ability of microalgae, which negatively impacts downstream process costs (Lu et al., 2016; Tricolici et al., 2014). A major challenge remains the low natural settling capacity of commonly used microalgae species, with harvesting representing one of the most energy-intensive steps and accounting for up to one-third of total production costs (Toh et al., 2014; Barsanti and Gualtieri, 2018; Fasaei et al., 2018). Unfortunately, the development of low-cost harvesting strategies is essential for sustainable microalgae cultivation. Granular activated algae systems have been proposed to improve biomass recovery by gravity sedimentation (Tiron et al., 2017); however, most current algaculture technologies rely on suspended cultures, requiring further advancements to improve scalability. Overall, these challenges are consistent with bottlenecks identified in reports prepared for the European Commission, which highlight contamination risks, high operational costs, downstream processing barriers, and the lack of updated datasets as key factors currently limiting the industrial application of microalgae-based technologies (European Commission, 2025b).

This study aimed to demonstrate an optimized biological treatment stage for dairy wastewater, based on suspended microalgae-bacteria biomass (hereafter referred to as *activated algae biomass*), operated in sequencing batch mode within a two-stage configuration under self-sustained aerobic conditions. The approach was designed as a nature-based alternative to conventional activated sludge technology, targeting efficient removal of organic matter and macronutrients while reducing hydraulic retention time. The research also investigated the influence of operational conditions on the current limitation in microalgae biotechnology, specifically the microalgae harvesting efficiency, and the ecological interactions within the operated activated algae system, with particular focus on protozoan communities as established bioindicators of biological wastewater treatment process stability.

## 2 Materials and methods

### 2.1 Origin of biomass and pre-adaptation

Microalgae biomass was obtained from internally preserved cultures originating from a laboratory-scale reactor treating dairy wastewater with activated sludge. The inoculum was represented by small-cell *Chlorella* sp. (<10 µm), characterized by a low settling velocity (<0.005 m/h), and an associated native bacterial community originating from the wastewater treatment operation. The biomass was subjected to a conditioning phase for re-adaptation to dairy wastewater. The acclimation lasted two months under controlled laboratory conditions to generate microalgae-bacteria consortia progressively adapted to different levels of dairy wastewater. No deliberate inoculation of additional bacteria or microalgae taxa was performed during the experiment.

During the first month, the biomass was cultivated in 250 mL Erlenmeyer flasks containing 120 mL Chlorella Broth medium and 30 mL raw dairy wastewater (80:20, v/v). At this stage, part of the biomass was maintained for an additional month under the same conditions, while another part was transferred to flasks with an adjusted composition of 90 mL Chlorella Broth and 60 mL dairy wastewater (60:40, v/v) and cultivated for one more month. Cultures were incubated in an orbital shaker (INNOVA^®^ 44R, New Brunswick Scientific, USA) at 25 ± 1 °C, under a 12:4 h light: dark photoperiod, with continuous agitation at 100 rpm. Half of the culture medium was replaced weekly to sustain nutrient availability.

### 2.2 Experimental set-up

#### 2.2.1 Activated algae production

The experimental configuration and operational adjustment of the biological wastewater treatment were defined based on key findings from previous in-house research:

- the use of microalgae-bacteria biomass for raw dairy wastewater treatment ensured, within a 24 h batch time, COD removal efficiencies above 80% while ammonium (NH_4_^+^) removal was consistently below 50% (Tricolici et al., 2014);
- COD loading strongly influenced the variation of oxygen saturation in the mixed liquor; although microalgae photosynthesis could sustain over-saturation, oxygen levels during sequencing batch operation could still decrease to 0% during the reaction phase under light exposure, and the duration of this depletion correlated directly with COD loading, implicitly with the activity of aerobic microorganisms involved in organic matter degradation (Tiron et al., 2015).

Based on these observations, the biological treatment process was split into two stages: Treatment Stage 1 (TS1) and Treatment Stage 2 (TS2). Before the experimental operation, both stages were used for the production and adaptation of activated algae biomass under stage-specific operational conditions.

##### 2.2.1.1 Treatment stage 1

**Figure 1:**
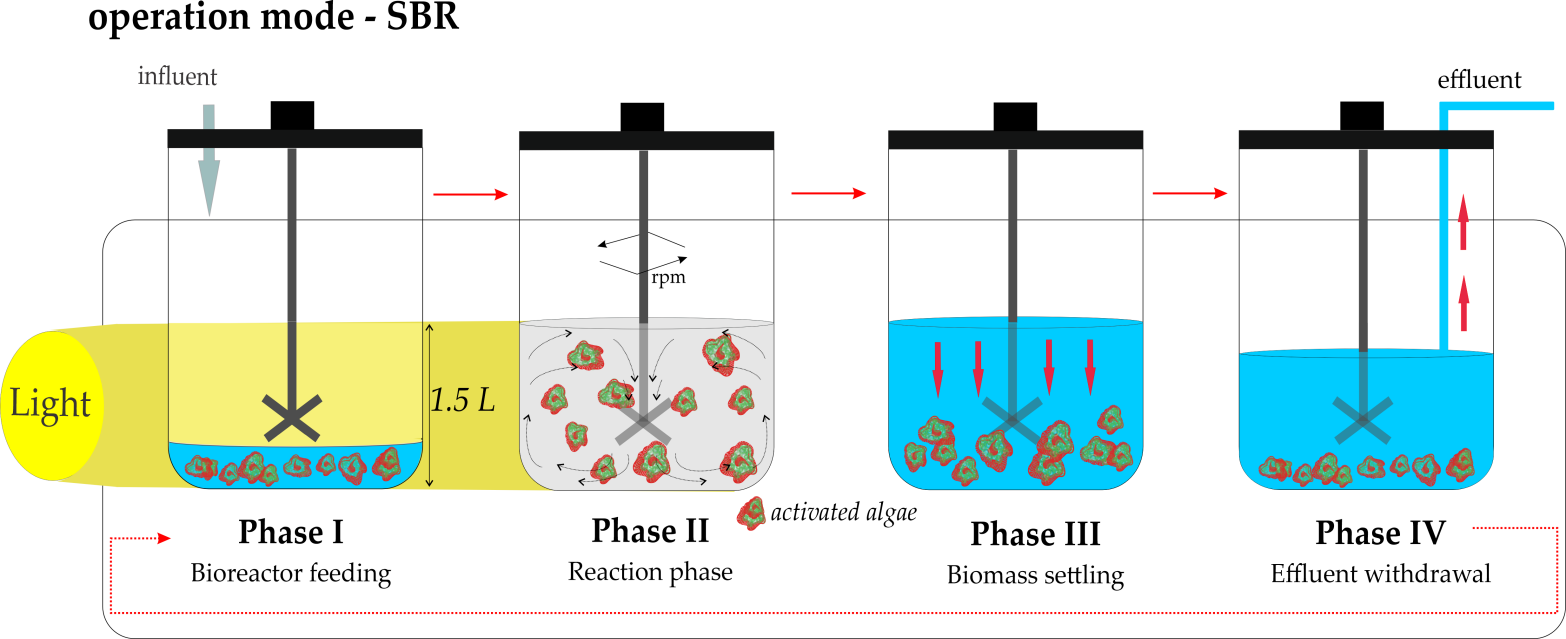
Schematic representation of the SBR operation during biomass culturing and dairy wastewater treatment in the case of TS1 and TS2 stages.

The first treatment stage (TS1) was designed to use activated algae biomass adapted to high organic loading (> 1 g O_2_L), typical for dairy wastewater, functioning as a self-sustained oxygen supply system relying solely on photosynthesis. The objective was to decrease COD below 0.5 g O_2_/L, the national discharge limit for influents discharged into sewerage systems and wastewater treatment plants (Normative NTPA 002, 2005). This stage was operated in the Biostat^®^Bplus Twin bioreactor (BR1) (Sartorius, Germany). Prior to the treatment stage, pre-adapted microalgae-bacteria inoculum enriched at a higher wastewater ratio to nutrient medium (60:40, v/v; see 2.1 Origin of biomass and pre-adaptation) was transferred to raw dairy wastewater medium and cultivated in sequencing batch mode (SBR) for about two months (18 batches). Operational conditions applied during biomass cultivation and dairy influent quality are listed in **Tables 1**, **2**, respectively. Schematic illustration of the sequencing batch operation is shown in **Figure 1**. The resulting activated algae biomass was used in the first treatment stage TS1 at an initial concentration of 0.6 ± 0.03 g/L.

**Table 1:** Operational conditions established during activated algae culturing under high and low COD loadings.

Operational conditions	Biomass adaptation for the first treatment stage –TS1	Biomass adaptation for the second treatment stage – TS2
Bioreactor type	BR1 - Biostat^®^Bplus Twin	BR2 – Biostat^®^Aplus
Vessel volume (*maximum*)	4 L	2 L
Working volume	1.5 L	1.5 L
COD loading	High strength wastewater (> 1 g O_2_/L)	Low strength wastewater (< 0.5 g O_2_/L)
Operation mode	SBR	SBR
Sequence time:	72 h (3 days)	72 h (3 days)
1 *Bioreactor feeding*	10 min	10 min
2 *Reaction phase/HRT*	71 h 10 min	71 h 10 min
*Stirring*	75 rpm	110 rpm
3 *Settling time*	30 min	30 min
4 *Effluent withdrawal*	10 min	10 min
Influent volume	1.2 L	1.2 L
Recycling rate%	20%	20%
Light intensity*	820 µmol m^-^² s^-^¹	820 µmol m^-^² s^-^¹
Photoperiodicity	15 h light/9 h dark	15 h light/9 h dark
Liquor pH control**	between 6.5 and 8.5 value	between 6.5 and 8.5 value

*At the outer wall of the vessel.

**with 0.1 N H_2_SO_4_ and 0.1 N NaOH.

**Table 2:** Physico-chemical properties of the dairy influent used for activated algae cultivation under high and low strength wastewater conditions (>1 g O_2_ COD/L for *Influent BR1* and<0.5 g O_2_ COD/L for *Influent BR2*, respectively), and during optimization of the biological treatment stage (*Influent TS1*).

Parameter	Measurement unit	Biomass culturing		Influent TS1
		Influent BR1	Influent BR2	
pH	–	7.02 - 7.30	6.97 - 7.19	6.93 - 7.26
O_2_	%	< 5	< 5	< 5
COD	g O_2_/L	1.408 - 2.860	0.145 - 0.383	1.230 - 1.716
NH_4_^+^	mg/L	35.4 - 68.9	19.9 - 26.3	21.9 - 69.9
NO_2_^-^	mg/L	< 0.1	< 0.1	< 0.1
NO_3_^-^	mg/L	< 0.1	< 0.1	< 0.1
PO_4_^3-^	mg/L	13.1 - 31.9	10.9 - 15.7	0.8 - 25.2
Mg^2+^	mg/L	16.2 - 36.8	10.2 - 15.6	20.6 - 28.2
Ca^2+^	mg/L	13.3 - 24.1	14.1 - 19.3	15.9 - 24.7

##### 2.2.1.2 Treatment stage 2

The second treatment stage (TS2) was operated in a Biostat^®^Aplus bioreactor (BR2) (Sartorius, Germany). The target of this stage was to increase ammonium and phosphate removal from TS1 effluents by using activated algae biomass adapted to low-strength influents with COD below 0.5 g O_2_/L. Since direct ammonium assimilation was insufficient to achieve high removal efficiency (Tricolici et al., 2014), the process design also focused on sustaining nitrification. This was addressed by targeting high oxygen saturation during the light phase for longer periods than in TS1 (>65% of the illuminated period), enabled by the reduced COD load. As in the case of Treatment Stage 1, a preliminary enrichment stage was carried out with microalgae-bacteria inoculum adapted at a lower dairy wastewater ratio to nutrient medium (80:20, v/v; see 2.1), cultivated in five to seven times diluted raw dairy wastewater to decrease COD below maximum allowed discharge limits for influents. Based on previous results (Tiron et al., 2015), it was expected that lower COD would promote, during the light phase, shorter oxygen-depletion periods at 0%, favoring the growth of aerobic bacteria, including ammonium oxidizers, known to co-exist with microalgae (Zhou et al., 2015). To maintain adequate macronutrient concentrations after dilution, supplementary NH_4_^+^, PO_3_^4-^, Ca^2+^, and Mg^2+^ were added (at concentrations: 45 mg/L NH_4_Cl, 15 mg/L K_2_HPO_4_, 30 mg/L CaCl_2_ · 2H_2_O, and 100 mg/L MgSO_4_· 7H_2_O, respectively). These concentrations were aligned with national limits for influent wastewater. Operational conditions set up during culturing are presented in **Table 1** and the resulting influent quality is listed in **Table 2**. Inoculum enrichment was performed during the 19 culturing batches. The resulting activated algae biomass was used in the second treatment stage TS2 at an initial concentration of 1.04 ± 0.07 g/L.

**Figure 2:**
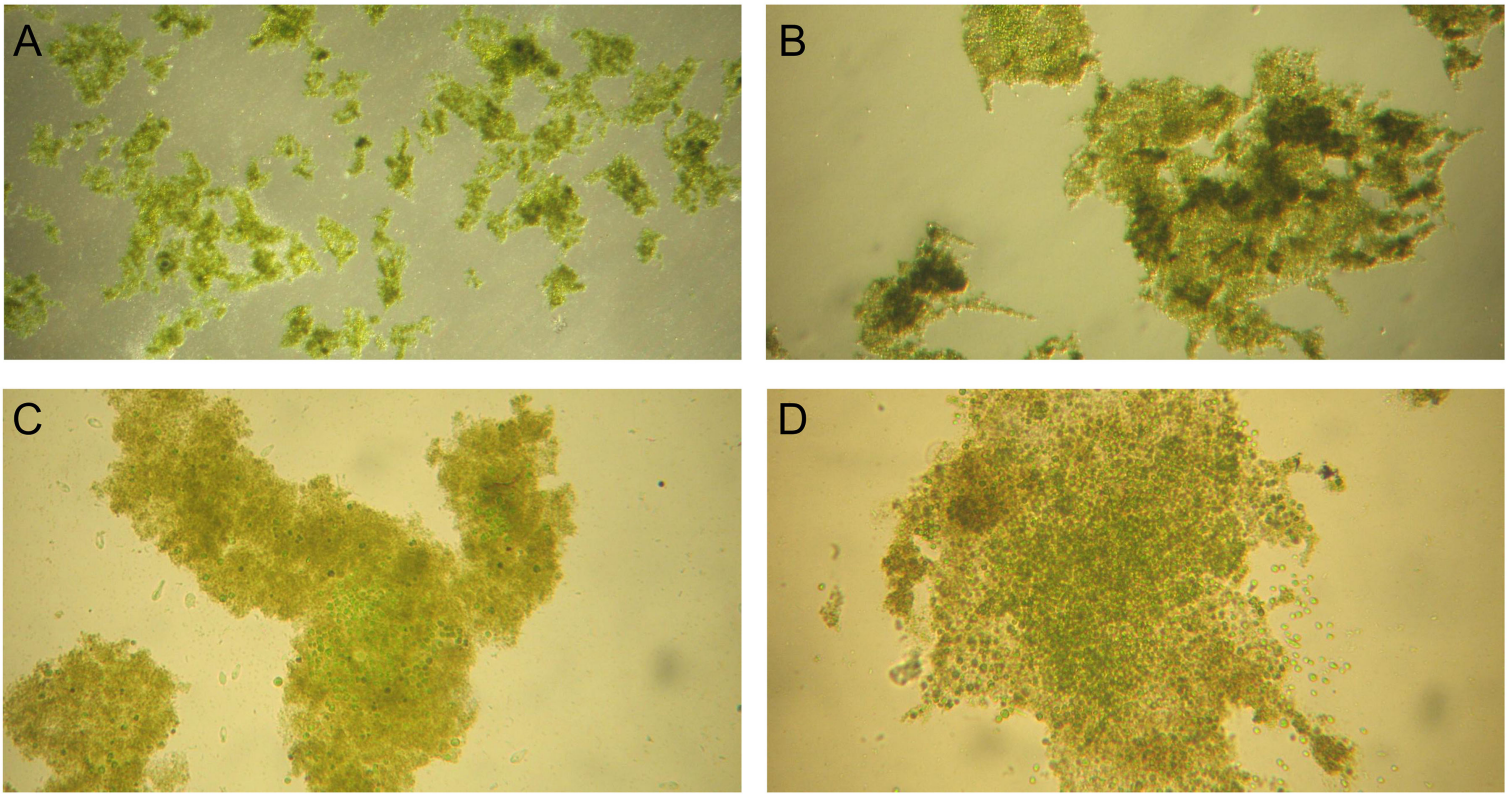
Microscopic observations of the activated algae biomass prior to experimental operation in Treatment Stage 1 (TS1). Magnifications: **(A)** 40×, **(B)** 100×, **(C)** 200×, and **(D)** 200×.

**Figure 3:**
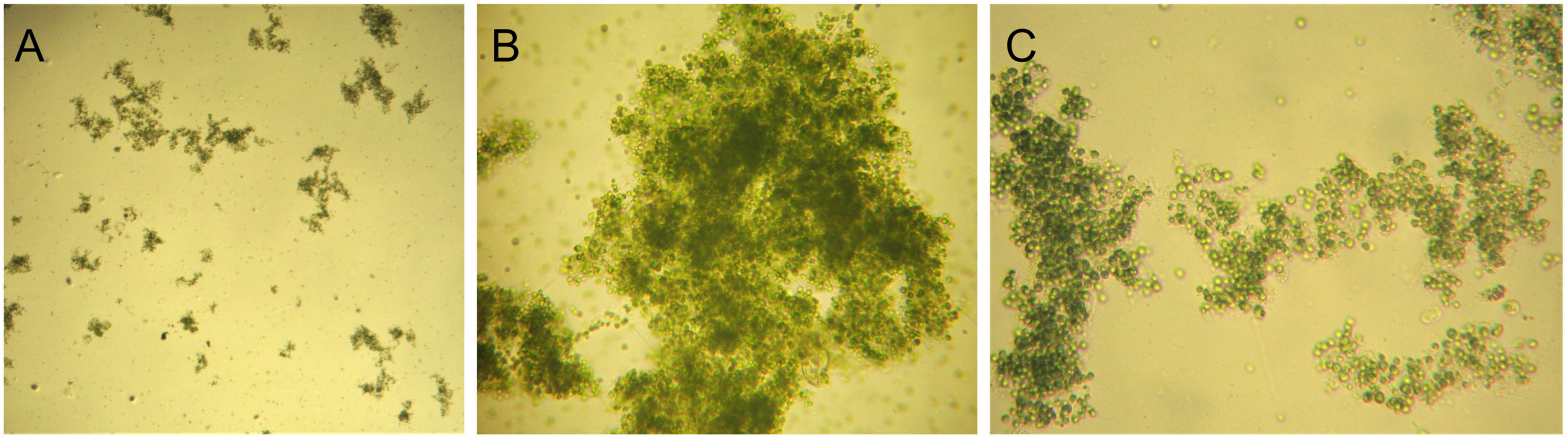
Microscopic observations of the activated algae biomass prior to experimental operation in Treatment Stage 2 (TS2). Magnifications: **(A)** 40×, **(B)** 200×, and **(C)** 200×.

For both stages, light intensity and HRT parameters were set according to previous studies (Tricolici et al., 2014; Tiron et al., 2015). After each effluent withdrawal, settled biomass was re-mixed with new influent, a condition applied to all performed culturing and treatment batches. pH was controlled and maintained below 8.5 value in accordance with national environmental regulatory framework Normative NTPA 001 (2005) and Normative NTPA 002 (2005), both to comply with influent/effluent limits (6.5–8.5) and to avoid ammonification and nutrient precipitation. The microalgae-bacteria biomass used to start dairy wastewater treatment stages displayed a heterogeneous structure, with suspended *Chlorella* sp. cells and floccular aggregates containing both microalgae and bacteria communities (**Figure 2**, **Figure 3**). Suspended microalgae cells significantly impacted effluent quality; effluents from both reactors contained abundant free cells, exceeding 75 µg chlorophyll *a*/L, typical of hypertrophic aquatic ecosystems, thus requiring a mandatory additional harvesting step prior to effluent discharging.

#### 2.2.2 Experimental operation

The optimized dairy wastewater treatment was conducted in the same two photobioreactors (BR1 and BR2) previously used for biomass enrichment, operated simultaneously in sequencing batch mode as illustrated in **Figure 1**. The experimental phase started directly with the batch following the 18 (BR1), respectively 19 (BR2) enrichment cycle, employing the *in situ* maintained activated algae biomass described in Section 2.2.1, without further re-inoculation. Operational conditions applied to BR1 (TS1) and BR2 (TS2) were identical to those established during biomass production (**Table 1**). The influent quality at the start of the experimental phase is summarized in **Table 2**, with *Influent TS1* representing the wastewater composition entering the first treatment stage. Effluent withdrawn from BR1, including unsettled biomass after the settling phase, was directly supplied as influent to BR2, resulting in a cascaded treatment configuration.

In both treatment stages, wastewater treatment was performed without mechanical aeration, relying exclusively on oxygen supplied by photoautotrophic microalgae. A total of 20 treatment batches were operated sequentially under this optimized flow configuration. Process monitoring included COD and macronutrient concentrations, dissolved oxygen, pH, and microalgae biomass recovery efficiency, which were assessed during 13 representative batches (B1-B13) from the total operated. The dairy wastewater used in the study was collected from the distribution channel of a dairy industry specializing in fresh dairy products and cheese.

The present study was designed as a process-oriented, exploratory investigation aimed at evaluating the effectiveness of an activated microalgae–bacteria system in sustaining efficient treatment performance while maintaining biological and operational stability under practice-oriented, non-sterile conditions. The experiments were conducted within a two-stage reactor configuration, with the experimental design focused on process behaviour and system functionality to provide preliminary data relevant for future optimisation and scale-up.

### 2.3 Assessment of microalgae biomass harvesting efficiency and activated algae concentration

Microalgae harvesting efficiency was calculated based on chlorophyll *a* concentrations according to the following (Equation 1)

(1) $$R\;\left(\%\right)=\frac{Chl_{i}-Chl_{f}}{Chl_{i}}\times 100\%$$

where R (%) is the harvesting efficiency, Chl_i_ (mg/L) is the chlorophyll *a* concentration in the homogenized liquor before the settling phase, and Chl_f_ (mg/L) represents the concentration in the effluent withdrawn after the settling phase. Chlorophyll *a* (mg/L) was determined spectrophotometrically according to SR EN 10260:1996 using a CLARIOstar microplate reader (BMG Labtech GmbH, Germany). Activated algae biomass concentration was determined as dry weight following the gravimetric method described in SR ISO 10260, 1996 (adapted for biomass samples); a defined volume of homogenized liquor was filtered through pre-dried and pre-weighed glass microfiber filters (Ø47 mm, Whatman GF/C type, Milli-pore, USA) using a vacuum/pressure pump (WP6122050, Millipore, USA). Filters were subsequently dried at 90 °C for 24 h, and biomass concentration was calculated as (Equation 2):

(2) $$D\;\left(g/L\right)=\frac{F_{90}-F_{0}}{V}\times 1000$$

where D (g/L) is the biomass concentration (dry weight), F_0_ (g) is the initial weight of the dried filter, F_90_ (g) represents the filter weight after drying with retained biomass, and V (mL) is the sample volume, and 1000 is the conversion factor from milliliters (mL) to liters (L).

### 2.4 Analytical methods

Dissolved oxygen (DO) and pH profiles were continuously monitored throughout the batch cycles. Measurements were performed using OxyFerm FDA sensors for DO and EasyFerm Plus K8 electrodes for pH (both Hamilton, Switzerland). The oxygen level was expressed as a percentage of saturation (%), where 100% corresponds to approximately 8.26 mg of dissolved oxygen per liter at the operating temperature of 25 ± 1 ^0^C. Chemical oxygen demand (COD) was analyzed according to SR ISO 6060, 1996. The concentrations of ammonium (NH_4_^+^), magnesium (Mg^2+^), and calcium (Ca^2+^) were determined following SR ISO 14911, 2003, while nitrite (NO_2_^-^), nitrate (NO_3_^-^), and phosphate (PO_4_^3-^) were analyzed according to SR EN ISO 10304-1, 2009 using an ICS-3000 ion chromatography system (Dionex, USA). COD and nutrient concentrations were measured at 24 h intervals during the reaction phase. Reported values are expressed as arithmetic means obtained from technical replicates (triplicate analyses for each sampling point). For each investigated treatment batch and sampling time, three independent analytical samples were analyzed, and the data are presented as mean ± standard deviation.

The size distribution of *Chlorella* sp. cells was investigated on preserved samples using the laser diffraction (Mastersizer 2000, Malvern Instruments, UK) with a particle refractive index of 1.060 (Aas, 1996). Biomass samples for microscopic investigations were collected during the reaction phase of each treatment batch, specifically within the final 24 h of the 72 h batch cycle. Microscopic investigations were conducted on biomass samples using an optical trinocular microscope (Model B1, Optech, Germany) to assess biomass structure, biological status, and dynamics. Major biological components, including microalgae, protozoa, and metazoans, were identified based on morphological characteristics, in selected cases where such features allowed reliable differentiation. The presence of bacterial biomass, as a complementary biological component to microalgae, was qualitatively assessed microscopically, without taxonomic identification. Microalgae and bacterial activity were evaluated indirectly through monitored analytical parameters, in accordance with established biological wastewater treatment operational practices (Henze et al., 2008).

## 3 Results

### 3.1 Dissolved oxygen profile

**Figure 4:**
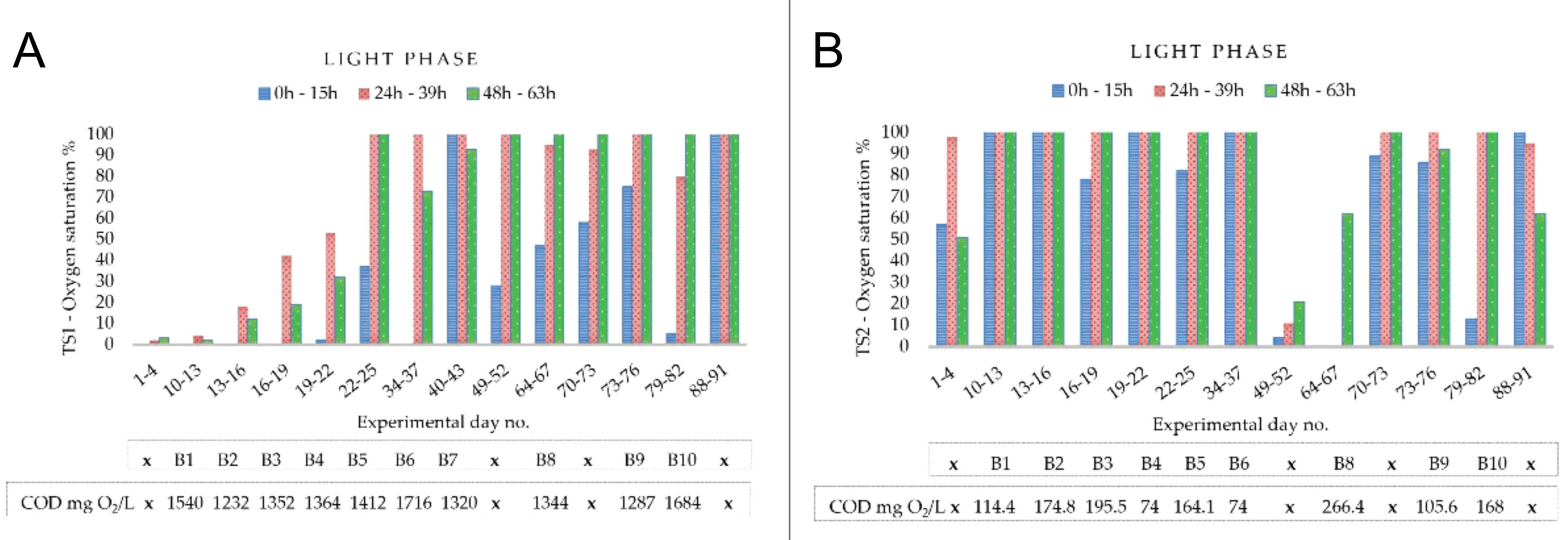
Variation of maximum oxygen saturation (%) recorded in the mixed liquor during dairy wastewater treatment: **(A)** TS1, **(B)** TS2. Each set of three consecutive experimental days corresponds to a 72 h batch. The batch number *Bn* and the corresponding influent COD concentration are indicated below the experimental days. The symbol *x* marks batches that were not targeted for full monitoring according to the experimental plan, for which only pH and oxygen were continuously recorded. Values correspond to direct sensor recordings.

Dissolved oxygen (DO) and pH were continuously monitored throughout the study in all conducted batches. Considering the established HRT (71 h 10 min) and the applied light: dark periodicity (15 h:9 h), maximum DO peaks were registered during 0–15 h, 24–39 h, and 48–63 h of each batch cycle. The corresponding DO dynamics for TS1 and TS2 treatment stages are presented in **Figures 4A, B**, respectively, reported for representative three-day experimental intervals corresponding to selected batches and correlated with influent COD concentrations.

#### 3.1.1 Oxygen dynamics under high organic loading (TS1)

In the first treatment stage, DO levels in the mixed liquor showed distinct dynamics depending on the influent organic load. At the highest COD concentrations, such as 1540 ± 82 mg O_2_L (days 10–13) and 1716 ± 96 mg O_2_L (days 34–37), oxygen saturation remained close to 0% throughout the first day, reflecting a strong dominance of bacterial oxygen demand over microalgae oxygen release. Under moderate COD loads in the range of 1200–1400 mg O_2_L, oxygen accumulation occurred earlier, with DO levels increasing to 18–53% within the first 24 h and frequently reaching supersaturation (>100%) by the third day. In other batches, such as at 1412 ± 85 mg O_2_L (days 22–25) and 1344 ± 77 mg O_2_L (days 64–67), oxygen exceeded 37–47% already within the first 15 h, pointing to a faster balance between oxygen production and consumption. Of particular note, at the COD level of 1320 ± 69 mg O_2_L (days 40–43) and in the later cycle (days 88–91), DO saturation surpassed 100% from the very beginning of the reaction phase, indicating that particularly favorable conditions enabled rapid oxygen accumulation despite the relatively high organic load. During dark periods, DO consistently decreased to 0%, confirming the complete reliance of the system on light-driven oxygen production.

#### 3.1.2 Oxygen dynamics under reduced organic loading (TS2)

The second treatment stage exhibited distinct oxygen dynamics compared to TS1. In most batches, DO increased and exceeded 50% already within the first 24 h, with frequent occurrence of supersaturation (>100%) during the second and third days of the cycle (24–39 h and 48–63 h, respectively). Since TS2 was fed with BR1 effluent, influent COD levels (as shown below the TS2 chart, **Figure 4B**) were markedly lower than raw influent entering TS1, reflecting an about 80–95% COD removal efficiency achieved in the first stage. This reduction in organic load decreased bacterial oxygen demand, supporting oxygen accumulation through photosynthesis. At high DO saturation levels during the light phase, particularly above 100%, values decreased by about 5–20% at the onset of the dark phase. This pattern extended aerobic conditions beyond the illuminated period. Consequently, TS2 maintained DO above 0% for longer periods, creating stable conditions for aerobic processes, including ammonium oxidation, as later demonstrated. Deviations from this general trend were observed in specific batches. In the Batch corresponding to days 49–52 (COD was not determined, marked “*x*”), DO remained low throughout the reaction phase, without exceeding 44%. In Batch B8 (days 64–67), influent COD reached 266.4 ± 16.9 mg O_2_L, higher than the typical range of 100–200 mg O_2_L, resulting in suppressed DO accumulation (0% during the first 24 h and a limited increase by the third day up to 62%). A similar, though less pronounced, effect occurred in Batch B9 (day 79–82), where influent COD was 168.0 ± 14.7 mg O_2_L and DO increased slowly (13% at 0–15 h) before recovering to supersaturation later in the cycle. In the later batch (days 88–91), DO was high from the onset (105% at 0–15 h) but decreased gradually during the cycle, stabilizing at 62% by 48–63 h, indicating a shift in oxygen dynamics under the given conditions.

### 3.2 COD and macronutrient removal efficiencies

#### 3.2.1 Performance of the first treatment stage

**Figure 5:**
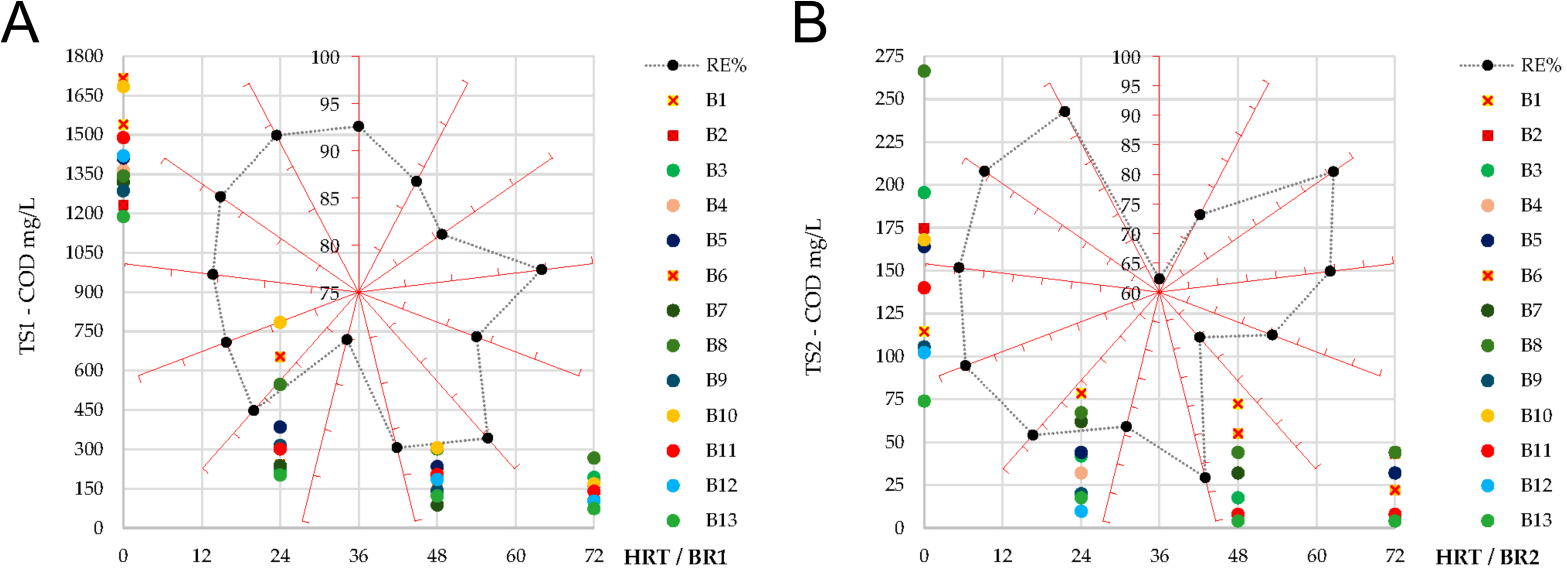
Variation of COD concentrations during sequential batch operation in the first (TS1 – BR1) and second (TS2 – BR2) treatment stages, and corresponding overall removal efficiency (RE%) achieved after each treatment stage: **(A)** TS1; **(B)** TS2. The RE% (black data points) are plotted on the red radial axes, corresponding to batches B1 to B13 in clockwise order starting from the top vertical axis. Values are reported as the mean of technical replicates (triplicate analytical measurements) performed at each sampling point.

In the first treatment stage, COD removal efficiency was consistently high, ranging from 80.2 ± 1.3% to 95.7 ± 0.7% across all monitored batches (**Figure 5A**). The decrease in COD concentration followed a similar trend in all cycles, with the most pronounced reduction occurring within the first 24–48 h of treatment, accounting for approximately 70–90% of the total COD removed in TS1. This achievement ensured suitable conditions for feeding BR2, with resulting effluent values varying between 0.074 ± 0.11 and 0.266 ± 0.017 g O_2_L.

**Figure 6:**
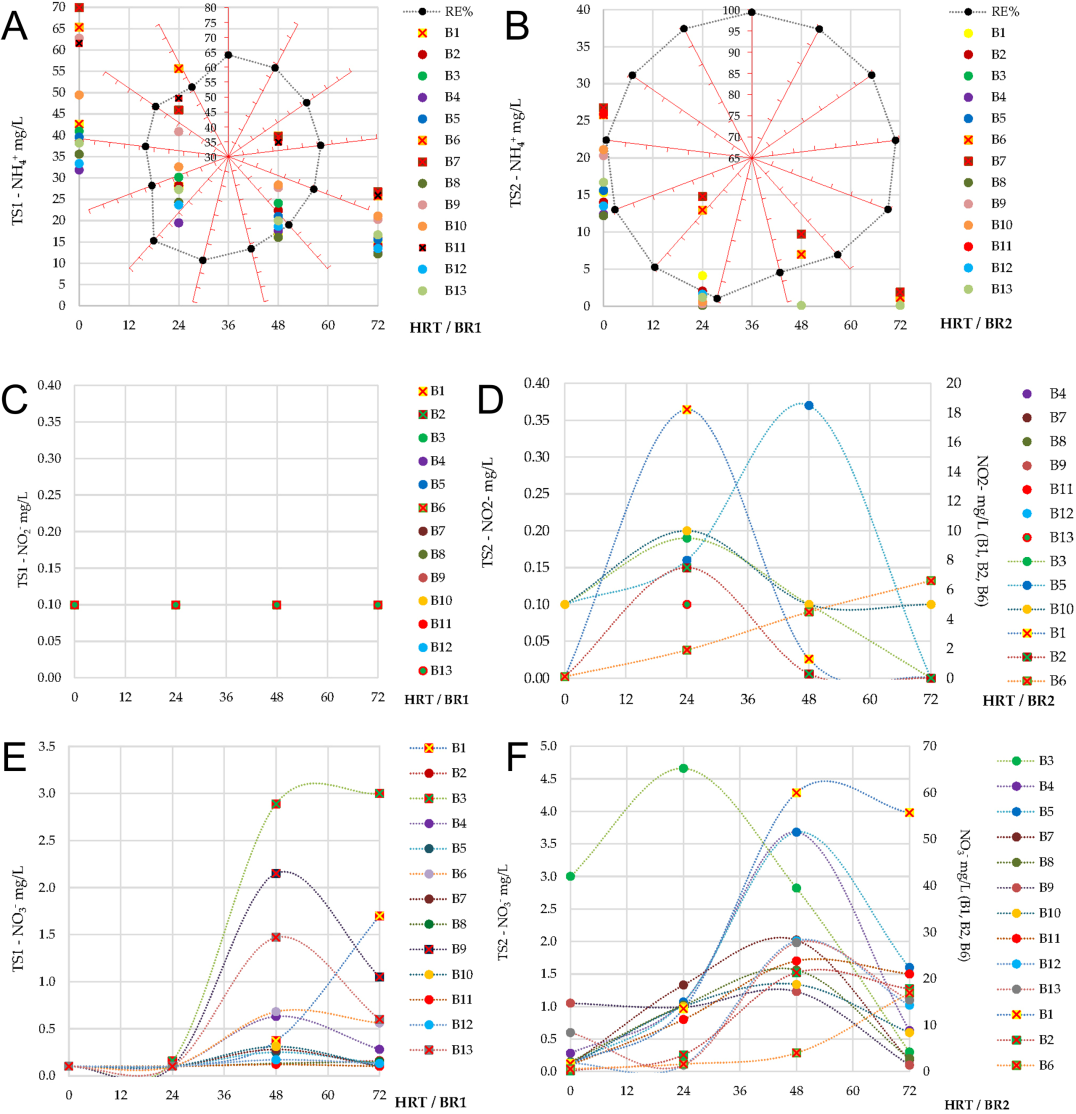
Ammonium (NH_4_^+^), nitrite (NO_2_^-^), and nitrate (NO_3_^-^) concentrations dynamics obtained during sequential batch operation in the first (TS1 – BR1) and second (TS2 – BR2) treatment stages: **(A, B)** variation of ammonium concentrations during operation and the corresponding overall removal efficiency (RE%) achieved after each treatment stage, where RE% values (black data points) follow a clockwise distribution from B1 (top axis) to B13; **(C, D)** variation of nitrite concentrations over the 0–72 h operational period for TS1 and TS2; **(E, F)** variation of nitrate concentrations over the 0–72 h operational period for TS1 and TS2. All values are reported as the mean of technical replicates (triplicate analytical measurements) performed at each sampling point.

Ammonium removal efficiency was moderate, ranging between 56.3 ± 1.6% and 67.7 ± 1.5% across batches (**Figure 6A**). Initial influent concentrations varied from 31.9 ± 0.8 to 69.9 ± 1.2 mg/L, with effluent values at 72 h between 12.2 ± 1.4 and 26.8 ± 1.9 mg/L. However, these concentrations remained below the maximum allowed limit for influents (< 30 mg/L), in relation to the national Normative NTPA 002 (2005). Despite episodes of oxygen supersaturation during the later part of the batches, the long periods of oxygen depletion (0%) constrained the persistence of nitrifying activity. Consequently, ammonium removal in TS1 was largely dominated by microalgae and bacteria assimilation processes, with only a limited contribution from nitrification, as further confirmed by the low nitrite and nitrate accumulation measured in effluents. Nitrite concentrations remained below the detection limit (0.1 mg/L) throughout the monitoring period, with no signs of accumulation under variable influent COD or NH_4_^+^ loads (**Figure 6C**). Nitrate concentrations remained generally low, without exceeding 3.0 ± 0.14 mg/L in the effluents. A gradual increase was observed during the batches, with NO_3_^-^ values rising after 24 h and peaking at 48–72 h (**Figure 6E**), consistent with the occurrence of nitrification under conditions of oxygen supersaturation.

**Figure 7:**
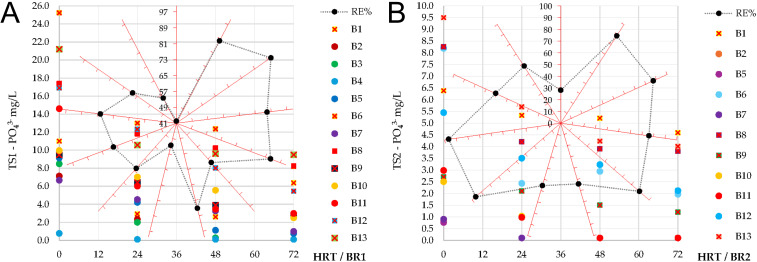
Variation of phosphate (PO_4_³^-^) concentrations during sequential batch operation in the first (TS1 – BR1) and second (TS2 – BR2) treatment stages, and corresponding overall removal efficiency (RE%) achieved after each treatment stage: **(A)** TS1; **(B)** TS2. RE% values are represented by black data points on the red radial axes, corresponding to batches B1–B13 in clockwise sequence. Values are reported as the mean of technical replicates (triplicate analytical measurements) performed at each sampling point.

Phosphate influent concentrations were highly variable, ranging from 0.77 ± 0.05 to 25.2 ± 0.64 mg/L. Removal efficiencies of this nutrient also fluctuated, from 42.0 ± 0.18% (B1) up to 98.8 ± 0.07% (B3), with final effluent values between below the detection limit (0.1 mg/L) and 9.5 ± 0.23 mg/L (**Figure 7A**), indicating a dependence on influent loading. Batches with higher initial concentrations (>15 mg/L; *e.g.*, B6, B8, B12, B13) generally showed lower removal (55–68%), while those with moderate influent values (<10 mg/L; *e.g.*, B2, B3, B4, B5) consistently achieved higher efficiency (>85%).

#### 3.2.2 Polishing performance of the second treatment stage

In the second treatment stage, COD removal performance was further improved compared to TS1. Overall efficiencies ranged between 62.2 ± 2.0% (B1) and 96.1 ± 0.2% (B10), with final effluent concentrations consistently below 50 mg O_2_L (**Figure 5B**). Expressed relative to the organic load received from TS1 effluents, this corresponded to an additional removal of 96.4 ± 1.1% to 99.7 ± 0.03%, confirming the polishing role of the second treatment stage.

Ammonium removal efficiencies were consistently high, above 92.9 ± 0.5% (**Figure 6B**). Effluent NH_4_^+^ concentrations were maintained below 0.1 mg/L in most batches, under the 2 mg/L discharge limit set by regulations. Occasional nitrite accumulation occurred in B1 (18.2 ± 0.3 mg/L at 24 h, decreasing to 1.3 ± 0.06 mg/L at 48 h), B2 (7.5 ± 0.2 mg/L at 24 h, decreasing to 0.3 ± 0.04 mg/L at 48 h), and B6 (6.6 ± 0.3 mg/L at 72 h). In all other batches, nitrite concentrations remained close to the detection limit (below 0.3 mg/L) (**Figure 6D**). These dynamics indicate that nitrifying activity was initiated in BR2, particularly under higher influent ammonium loads, but nitrite accumulation was temporary and followed by conversion to nitrate or assimilation within the biomass. Also compared with TS1, nitrate accumulation was more pronounced, supporting the occurrence of active nitrification. The highest effluent concentrations were recorded in B1 and B2 (55.7 ± 2.2 mg/L and 17.8 ± 0.9 mg/L, respectively), while in later cycles nitrate rarely exceeded 5 mg/L and in the final effluents generally remained close to the detection limit (**Figure 6F**), thus being below the 25 mg/L regulatory threshold (Normative NTPA 001, 2005).

Phosphate influent concentrations in TS2 ranged from<0.1 to 9.5 ± 0.23 mg/L. The recorded removal efficiencies varied between 28.1 ± 0.47% (B1) and 96.6 ± 0.04% (B10–B11), with final effluent concentrations between below the detection limit and 5 mg/L (**Figure 7B**). Most batches achieved efficiencies higher than 80%, although performance declined in B8 (54 ± 2.2%), B12 (61 ± 1.1%), and B13 (58 ± 1.8%), all characterized by higher influent PO_4_³^-^ levels. Compared with TS1, TS2 ensured lower residual phosphate concentrations, confirming its complementary role in achieving reliable nutrient removal.

#### 3.2.3 Overall treatment performance

**Figure 8:**
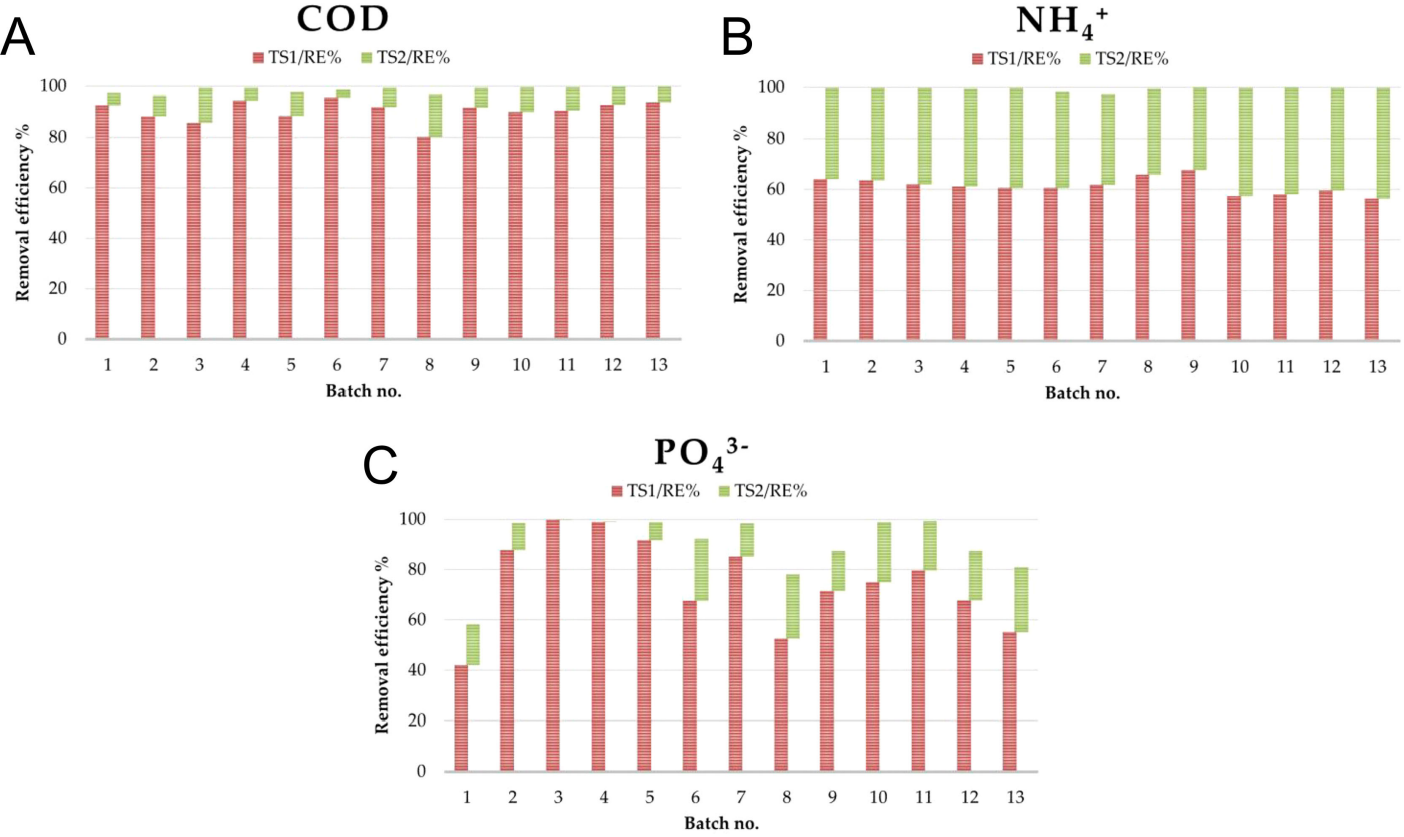
Overall removal efficiency (RE%) obtained for the monitored parameters during sequential batch operation in the first (TS1) and second (TS2) treatment stages: **(A)** COD; **(B)** ammonium (NH_4_^+^); **(C)** phosphate (PO_4_³^-^). Removal efficiencies were calculated based on arithmetic mean values obtained from technical replicates (triplicate analytical measurements) at each sampling point.

When considering the integrated performance of the two-stage treatment, the sequential system met the study objective of achieving simultaneous removal of organic matter and targeted macronutrients. Global COD removal ranged between 96.4 ± 1.1% (B2) and 99.7 ± 0.03% (B12–B13), with an overall average of 98.9% (**Figure 8A**). As a result, residual COD concentrations in the final effluents were below 50 mg O_2_L, in compliance with the national discharge limit of 125 mg O_2_L (Normative NTPA 001, 2005). The sequential design demonstrated its advantage by compensating for variability in TS1, particularly in batches with lower initial COD removal, where the polishing step of TS2 secured final high efficiencies. For ammonium, global removal efficiency exceeded 97% in all monitored batches, varying from 97.3 ± 0.28% (B7) up to 99.8 ± 0.06% (B12–B13) (**Figure 8B**). Final effluent NH_4_^+^ concentrations were generally below the detection limit (<0.1 mg/L). In the case of phosphate, global performance showed higher variability compared with COD and ammonium, ranging between 58.3 ± 1.47% and 99.5 ± 0.3% (**Figure 8C**). Final effluent phosphate concentrations remained below 5 mg/L in most cycles.

### 3.3 pH

**Figure 9:**
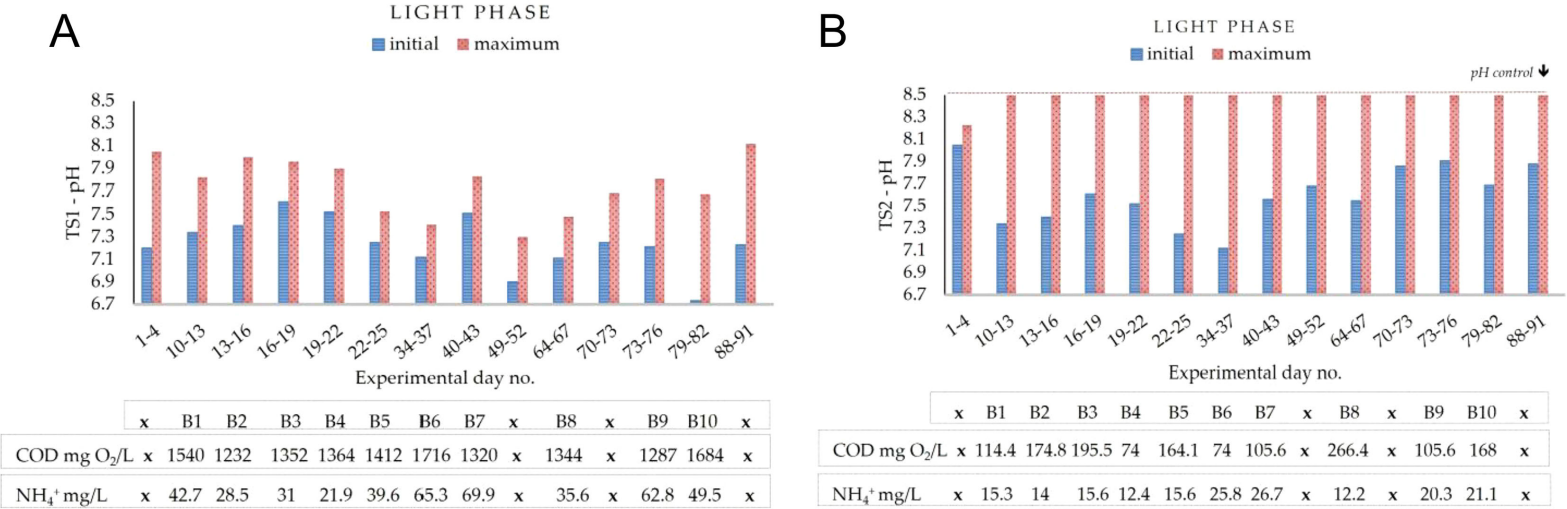
Variation of pH in the mixed liquor during dairy wastewater treatment. **(A)** First treatment stage (TS1); **(B)** Second treatment stage (TS2). For each batch, the initial (influent) and maximum in-cycle pH values are shown, together with the corresponding influent COD and ammonium concentrations. Values correspond to direct sensor recordings.

**TS1.** In the first treatment stage, influent pH values ranged between 6.7 and 7.6, while the maximum in-cycle values did not exceed 8.1 (**Figure 9A**). The observed increase of approximately one unit reflects a moderate alkalinization of the mixed liquor, primarily driven by CO_2_ uptake during photosynthetic activity in the light phase. However, this effect was counterbalanced by the intense aerobic degradation of organic matter at high COD loads, which acted as a buffering mechanism and limited further pH rise. Overall, the moderate variability of pH confirmed that bacterial respiration exerted a dominant influence on the system, with photosynthesis ensuring stability within the regulatory range (6.5–8.5).

**TS2.** In the second treatment stage, influent pH values were slightly higher (7.1–8.0), while the maximum continuously stabilized at 8.5 from the second monitored batch onwards (**Figure 9B**). Compared to TS1, these results reflect the stronger influence of photosynthetic activity under reduced organic loading, promoting CO_2_ uptake originating exclusively from the biodegradation of organic matter and medium alkalinization. Although nitrification activity was evidenced in TS2, its acidifying effect was counterbalanced by the higher intensity of photosynthetic CO_2_ assimilation, resulting in a net increase of pH towards the upper threshold. Once this threshold of 8.5 was reached, the pH control system was automatically activated by dosing 0.1 N H_2_SO_4_, thereby preventing further increases. During dark phases, pH decreased by up to 0.5 units, highlighting the direct relation between light-driven photosynthetic activity and pH dynamics.

### 3.4 Microscopic observations of biomass and community structure

#### 3.4.1 Structural evolution of the biological community in the second treatment stage

**Figure 10:**
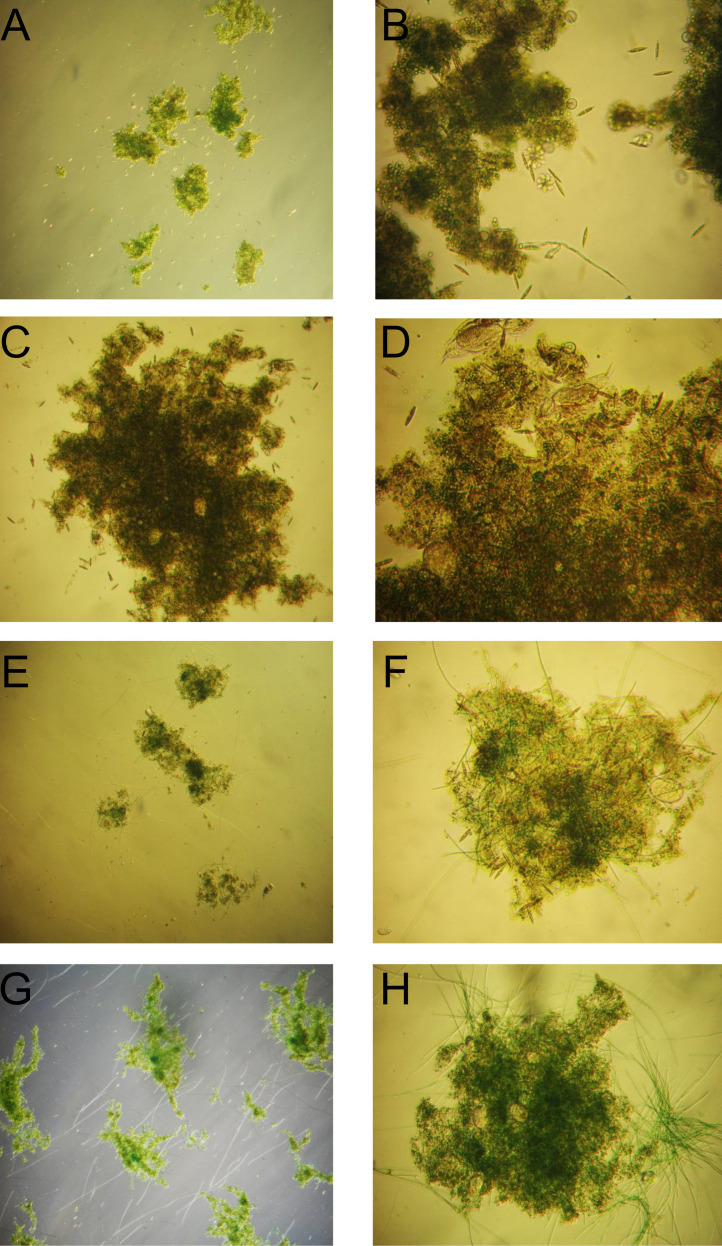
Microscopic observations in BR2 (TS2) across the batch series. Progressive rise of pennate diatom morphotypes and darkened aggregates: **(A)** 40x Batch 2; **(B)** 100x Batch 2; **(C)** 100x Batch 3; **(D)** 200x Batch 3. Emergence and proliferation of filamentous cyanobacteria phototrophs with reduced diatom frequency: **(E)** 100x Batch 6; **(F)** 200x Batch 6; **(G)** 100x Batch 8. Stable mixed consortia of *Chlorella* cells and filamentous phototrophs forming cohesive flocs: **(H)** 100x Batch 13.

Microscopic observations revealed a progressive diversification of the microalgae community in the second treatment stage. At the start of TS2, the biomass was dominated by unicellular green microalgae *Chlorella* sp. Beginning with monitored second batch (B2), diatom frustules (elongated, naviculoid/pennate forms) became visible (**Figures 10A, B**) and their frequency increased in Batch 3, where their elevated occurrence resulted in darker-pigmented flocs, reflecting high density within the phototrophic community (**Figures 10C, D**). From Batch 6 onward, non-toxic filamentous cyanobacteria morphotypes appeared (**Figures 10E, F**) and progressively increased in abundance, accompanied by a decline in diatom frequency (**Figure 10G**). In subsequent cycles (up to and beyond Batch 13), a gradual reorganization of the biomass structure was observed, with aggregates becoming more compact as *Chlorella* cells intertwined with filamentous phototrophs (**Figure 10H**).

**Figure 11:**
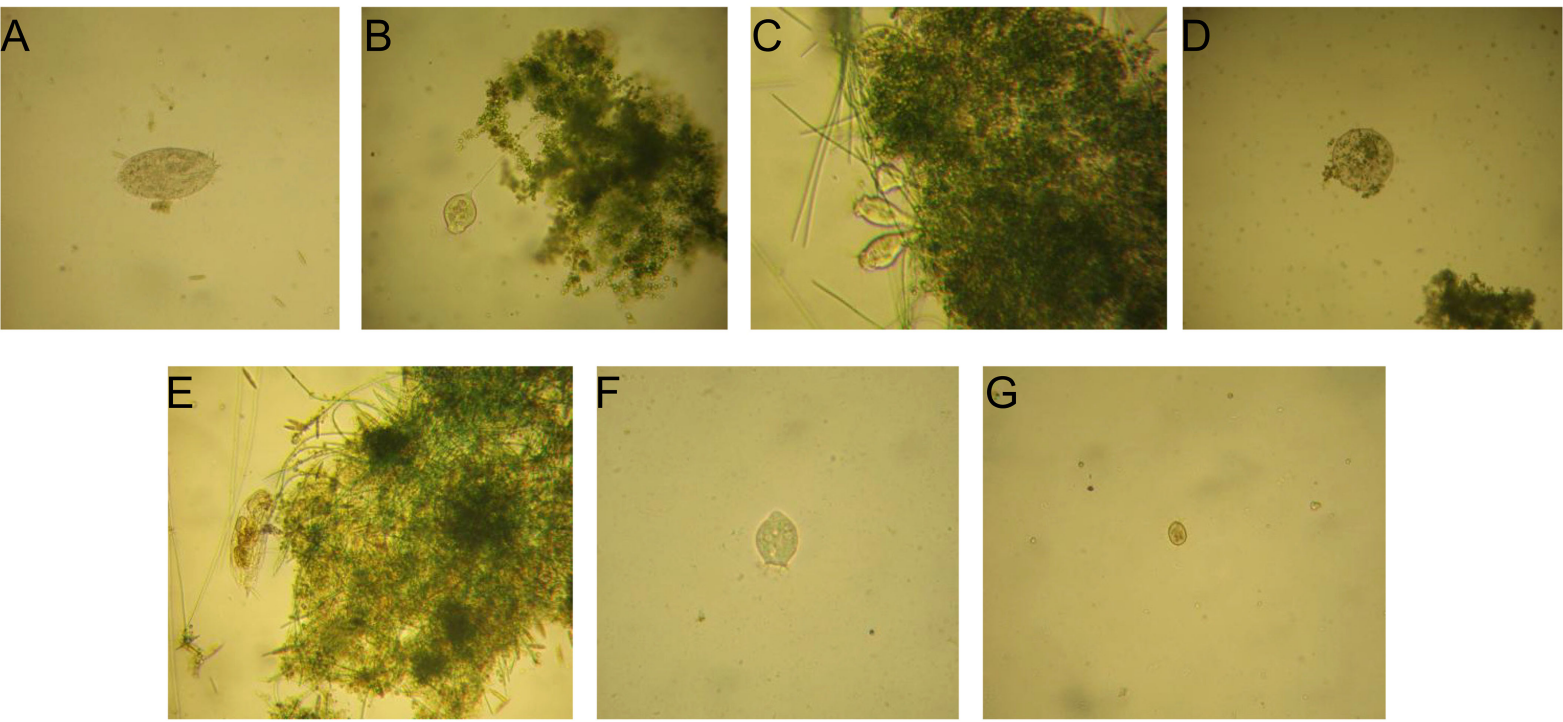
Microscopic observations of the protozoan and metazoan community identified in BR2 (TS2): **(A)** crawling ciliates *Urostyla* sp. 200x; **(B)** sessile attached ciliates *Vorticella* sp. 400x; **(C)** colonial stalked ciliates *Epistylis* sp. 400x; **(D)** testate amoebae *Arcella* sp. 200x; **(E)** rotifers 200x, and in BR1 (TS1): **(F)** small free-swimming ciliates *Colpidium* sp. 200x; **(G)** sessile ciliates *Vorticella* sp. (non-attached form) 400x.

In parallel, a diverse protozoan and metazoan community, typical of conventional activated sludge systems, was identified. Protozoa included small free-swimming ciliates *Colpidium* sp. (similar to those identified in TS1 and likely carried over by decantation), crawling ciliates such as *Urostyla* sp. (**Figure 11A**), sessile ciliates (*Vorticella* sp.) in both attached (**Figure 11B**) and non-attached forms, alongside colonial stalked ciliates *Epistylis* sp. (**Figure 11C**). Testate amoebae (*Arcella* sp.) were also present (**Figure 11D**). Metazoan representatives, particularly rotifers (**Figure 11E**), became increasingly abundant in later batches, with numerous rotifer eggs recorded towards the end, indicating active reproduction and supporting the establishment of a stable and diversified biomass.

#### 3.4.2 Organization of the biological community in the first treatment stage

Microscopic observations of the first treatment stage revealed darker aggregates, with bacterial populations appearing more prominent than in TS2. The overall structure suggested a biomass largely shaped by heterotrophic activity, consistent with the strong COD removal efficiencies recorded during this stage. The microalgae community was represented by *Chlorella* sp., without evidence of additional phototrophic groups throughout the study. Protozoan diversity was lower compared to TS2, being restricted to small free-swimming ciliates *Colpidium* sp. (**Figure 11F**) and occasional sessile ciliates of the genus *Vorticella* (non-attached forms) (**Figure 11G**), reflecting a less structured trophic network associated with this stage.

### 3.5 Microalgae recovery efficiency

**Figure 12:**
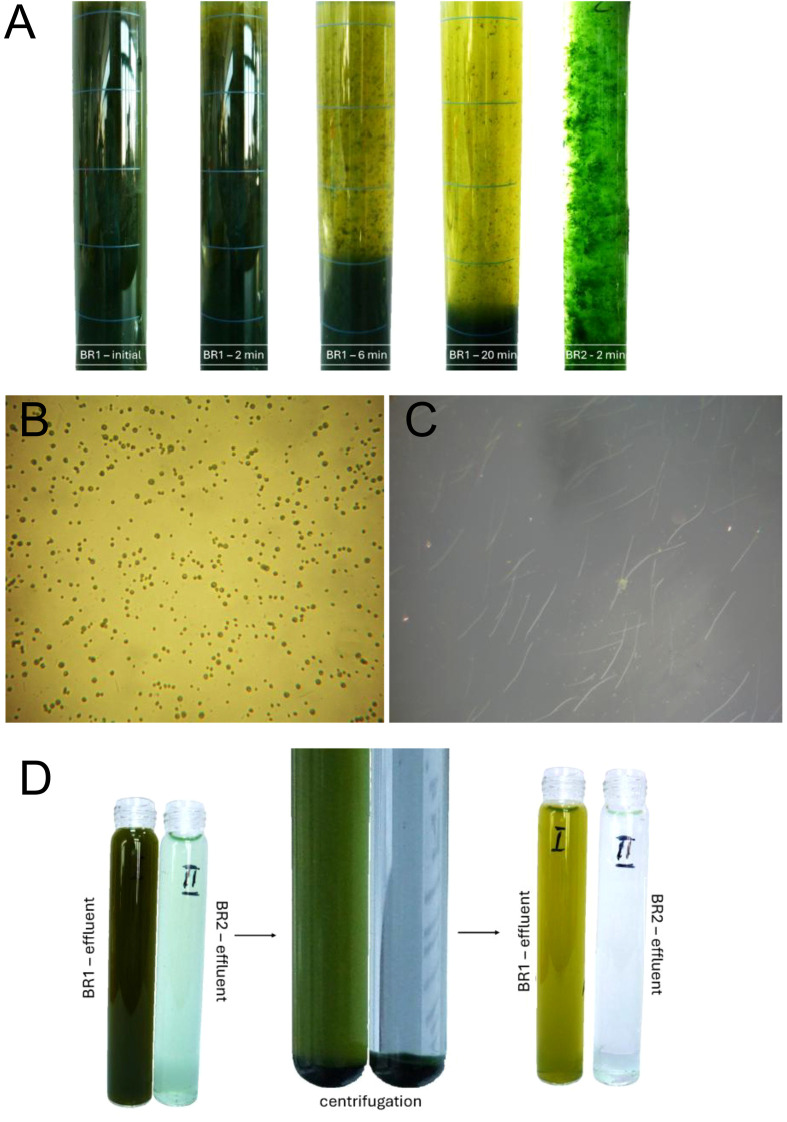
Microalgae recovery efficiency in the first (TS1 – BR1) and second (TS2 – BR2) treatment stages: **(A)** effluents recorded during the settling step at different time intervals (BR1: initial, after 2, 6, and 20 min; BR2: after 2 min); **(B)** microscopic view of suspended *Chlorella* sp. cells in BR1 effluent 400x; **(C)** microscopic view of effluent from TS2 showing reduced free-cell abundance of *Chlorella* sp. and prevalence of filamentous microalgae 40x; **(D)** improved biomass recovery after centrifugation (5000 rpm, 20 min).

Microalgae recovery efficiency recorded after the biomass settling step, in the first treatment stage, ranged between 76.2 ± 2.8% and 83.3 ± 2.3%. Despite these relatively high values, the resulting effluents retained a visible green coloration (**Figure 12A**), caused by suspended microalgae cells that remained after sedimentation. Subsequent centrifugation of the effluents at 5000 rpm for 20 min increased recovery to a maximum of 88.6 ± 2.7% (**Figure 12D**).

In the second treatment stage, recovery efficiency further improved, reaching 94.4 ± 1.8% after the settling phase. Compared to TS1, complete removal of microalgae suspended biomass was achieved after centrifugation (**Figure 12D**). Notably, the BR2 photobioreactor was fed not only with effluent from TS1 but also with unsettled biomass remaining after the TS1 settling step. As a result, BR2 was systematically inoculated at each feeding cycle with free *Chlorella* cells carried over from TS1. Nevertheless, recovery efficiency after TS2 exceeded that of the first stage.

Microscopic observations confirmed the high prevalence of free *Chlorella* sp. cells in TS1 effluents after sedimentation (**Figure 12B**), whereas in TS2 these free cells were markedly less frequent (**Figure 12C**), reflecting improved floc formation and enhanced biomass recovery.

## 4 Discussions

The dual role of microalgae in wastewater treatment and resource recovery has been increasingly highlighted. Beyond their ability to assimilate nutrients, microalgae act as efficient sinks for pollutants, providing a biological pathway for carbon capture along with nutrient removal. Importantly, the biomass generated during treatment can be harnessed into biofuels, fertilizers, or other high-value products, closing the loop between pollution mitigation and resource recovery (Arora et al., 2021; Ramírez Mérida and Rodríguez Padrón, 2023). Such integration positions microalgae-based systems as promising alternatives to conventional wastewater treatment technology, with the additional benefit of creating new value chains from residual biomass (Srimongkol et al., 2022). While much of the existing research has focused on cultivation strategies designed to minimize contamination, such approaches often rely on sterilized or synthetic wastewater or on influents and effluents with strongly reduced bacterial presence. These conditions cannot capture the complexity of full-scale treatment systems, where bacterial biomass can reach several grams per liter and cross-contamination is unavoidable, including within microalgae communities. This discrepancy limits the scalability of microalgae-based processes for real wastewater applications, particularly when applied directly in biological treatment stages, where the influent contains not only inorganic pollutants but also significant organic matter. This aspect is particularly relevant in the case of dairy wastewater, which is recognized as one of the most polluting industrial effluents, characterized by high chemical oxygen demand (COD) and suspended solids concentrations, making its discharge, when untreated or insufficiently treated, a significant threat to aquatic ecosystem health (Tocchi et al., 2013; Singh et al., 2024; Álvarez-Montero et al., 2025).

The present study addressed this challenge by operating with raw dairy wastewater, where bacterial populations were consistently present, and the organic matter and nutrient contents even exceeded the maximum discharge limits for sewerage networks and wastewater treatment plants. Using *Chlorella* sp., one of the most common microalgae taxa applied worldwide, provided an additional advantage, as it is widely recognized for its adaptability to diverse wastewaters and its established potential in biomass valorization pathways (Araújo et al., 2021).The oxygen and pH dynamics recorded in the experiments highlight the fundamental operational differences between the microalgae–bacteria system and conventional activated sludge biomass. In the absence of mechanical aeration, oxygen supply relied exclusively on the photosynthetic activity of photoautotrophic microalgae and was rapidly consumed by aerobic bacteria involved in organic matter degradation and nitrification. In TS1, dissolved oxygen remained at 0% during extended sub-periods, particularly under high organic loads, yet aerobic processes were not entirely suppressed, as oxygen micro-gradients at floc surfaces can sustain metabolic activity even under bulk liquor depletion (de Godos et al., 2014). The gradual rise in DO peaks observed after the first weeks reflected both adaptation to the imposed solids retention time and HRT regime and the decreasing heterotrophic oxygen demand as COD declined. In contrast, in TS2, the reduced organic load promoted faster DO accumulation, which even persisted into the dark phase. This provided an important operational advantage by sustaining aerobic conditions for ammonium oxidation without mechanical aeration. pH profiles corroborated this interpretation: while increases in TS1 were moderate (<8.1), reflecting the effect of intensive organic matter degradation, pH in TS2 consistently reached 8.5 value, driven by photosynthetic CO_2_ uptake at significantly lower COD loads. Although nitrification in TS2 exerted an acidifying influence, this effect was counterbalanced by photosynthesis, underlining the dominant role of phototrophs in shaping system alkalinity.

A comprehensive review by Singh et al. (2024) highlighted that most microalgae-based and microalgae-bacteria systems applied to dairy wastewater treatment rely on externally controlled operating conditions, particularly mechanical aeration and active pH regulation, often supported by CO_2_ supplementation, which also contributes to microalgae growth. While reported configurations can also achieve high nutrient and organic matter removal efficiencies, they remain dependent on energy- and resource-intensive inputs, recognized as major constraints for large-scale implementation.

Although microalgae-based systems are typically operated under moderate light intensities (generally below 400 µmol m^-^² s^-^¹) to prevent photoinhibition and oxidative stress (Richmond, 2013), the system in this study successfully operated at a higher irradiance of 820 µmol photons m^-^² s^-^¹. This is attributed to the mixed consortium structure, where the coexistence of phototrophic microalgae and bacteria influences photon distribution and utilization across the liquor, thereby mitigating the risk of localized overexposure. Moreover, the applied light intensity corresponds to approximately half of the average solar irradiance incident on the Earth surface under clear sky conditions (around 1800 µmol photons m^-^² s^-^¹; Kirk, 2011). Therefore, the light conditions applied in this study demonstrate feasibility for outdoor or pilot-scale implementation, allowing for optimization through reactor configuration and light distribution control, by case.

The sequential configuration proved highly effective for the removal of the targeted pollutants, ensuring compliance with discharge limits set by national environmental regulations. In TS1, COD removal efficiencies exceeded 80% across all monitored cycles, with most of the decrease occurring within the first 24–48 h. However, ammonium removal was more moderate (56–68%), primarily driven by assimilation rather than nitrification, as confirmed by the absence of nitrite accumulation and the limited nitrate concentration detected in effluents. Phosphate removal in TS1 was highly variable, reflecting the dependence of uptake on influent concentrations and the occurrence of saturation effects at higher loads. In contrast, TS2 acted as a polishing step, consistently lowering COD below 50 mg O_2_L, reducing ammonium to near-complete removal (>97%, often below the detection limit), and ensuring phosphate concentrations in effluents were generally below 5 mg/L. The occurrence of transient nitrite peaks and nitrate accumulation confirmed that nitrification was more active in TS2, supported by longer persistence of aerobic conditions and reduced COD competition.

When compared with recent studies employing integrated microalgae-bacteria systems or microalgae-dominated cultivation approaches for dairy wastewater treatment, the observed performance highlights contrasting removal performance patterns in the balance between organic matter and ammonium removal. For instance, microalgae-bacteria biofilm photobioreactors treating dairy manure wastewater achieved COD removal efficiencies of 85–97.4%, while NH_4_^+^-N removal was limited to 46.5% in the initial cycle and declined to approximately 18% in subsequent cycles, indicating instability of nitrogen removal under repeated operation (Chu et al., 2025). In contrast, microalgae-based cultivation systems operating in real dairy wastewaters reported NH_4_^+^-N removal exceeding 95% at early stages, but comparatively lower and variable COD removal of approximately 63–77%, partly associated with dissolved organic matter release under microalgae stress conditions (Cao et al., 2024). By comparison, the staged configuration applied in the present study enabled a progressive differentiation of dominant functional pathways, with TS1 supporting efficient COD reduction under high organic loading (80.2 ± 1.3 – 95.7 ± 0.7% COD removal) and TS2 providing stable and effective ammonium polishing (above 92.9 ± 0.5% NH_4_^+^ removal), resulting in global efficiencies of 96.4 ± 1.1 - 99.7 ± 0.03% for COD and higher than 97% for NH_4_^+^. This comparison indicates that process staging can overcome the limitations associated with balancing organic matter and ammonium removal reported for single reactor systems. Together, the two stages complemented each other: TS1 provided efficient organic load reduction, while TS2 ensured stable polishing and compliance with discharge limits.

Importantly, review analyses have emphasized that bacterial presence is often regarded as a limiting factor in microalgae-based dairy wastewater treatment, with most mitigation strategies focusing on bacterial suppression rather than functional integration (Singh et al., 2024). In contrast, the present results demonstrate that appropriately staged microalgae-bacteria coexistence can support stable treatment performance. This configuration enables efficient organic matter degradation and sustained ammonium removal without external aeration or CO_2_ supplementation.

Microscopy investigations further supported these findings by linking microbial structure with functional performance. In TS1, aggregates appeared darker and were more strongly associated with bacterial populations, while *Chlorella* sp. was present, and protozoan diversity was limited to small ciliates and *Vorticella* sp. This reflected a less complex trophic network, shaped by high organic load and bacteria-driven activity sustained aerobically by oxygen supplied from microalgae. In contrast, TS2 exhibited a gradual diversification of the phototrophic community, starting from flocs dominated by *Chlorella* in the first batches, with diatoms becoming increasingly frequent in B2–B3, and later evolving towards consortia where filamentous microalgae became prominent from B6 onward. The emergence of filamentous taxa coincided with longer solids retention time, lower dissolved COD, and recurrent oxygen supersaturation, conditions that favored their establishment and enhanced floc cohesion. Their co-occurrence with unicellular *Chlorella* species provided both rapid nutrient removal and structural stability, explaining the persistence of aerobic conditions into the dark phase and the improved polishing performance observed in TS2. The positive effect of microalgae community diversification on system stability and functional resilience has also been documented in the literature (Stockenreiter et al., 2016).

These changes in community structure also explain the observed differences in microalgae recovery performance. In TS1, suspended *Chlorella* sp. cells persisted after sedimentation, leading to green–brown effluents and requiring centrifugation to improve recovery. Although harvesting efficiency improved after centrifugation, the resulting effluent still contained residual suspended microalgae, indicating the need for a more intensive or alternative biomass recovery approach to achieve full clarification.

**Figure 13:**
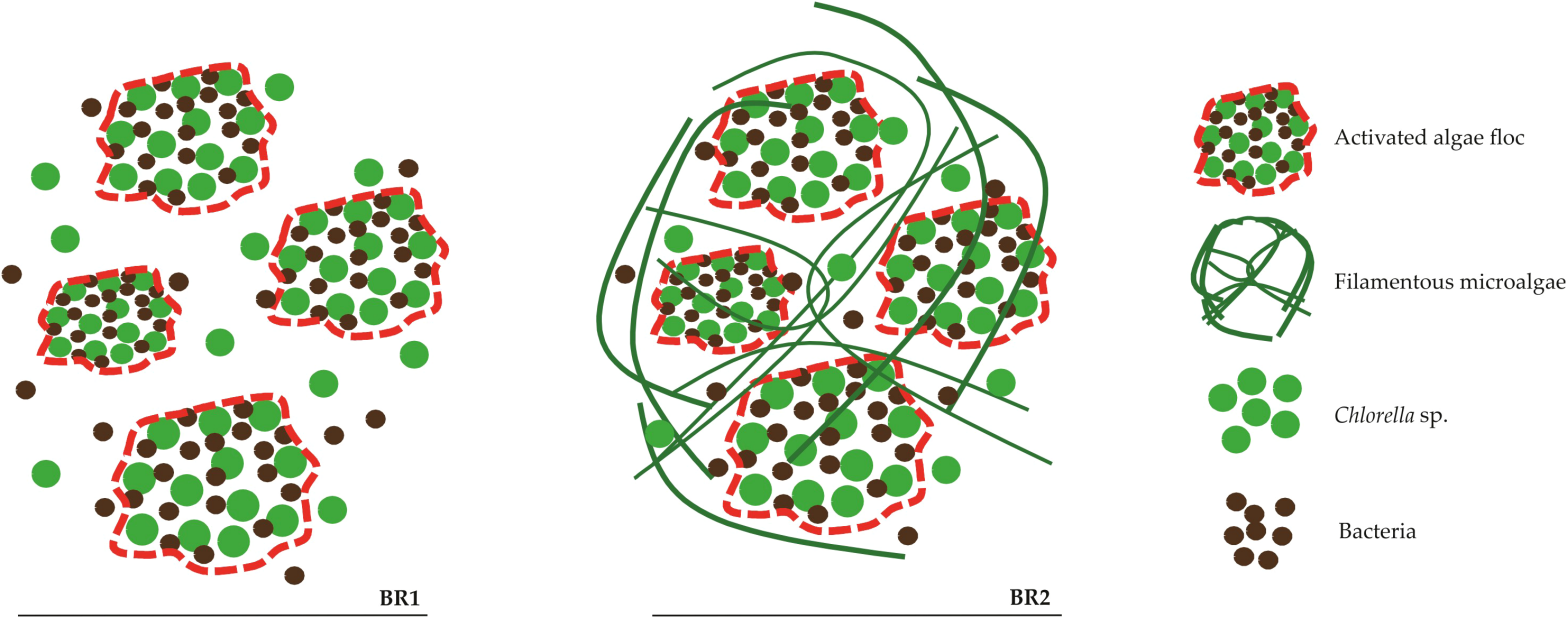
Schematic representation of biomass organization in BR1 (TS1) and BR2 (TS2). In BR1, activated algae flocs were smaller and surrounded by free *Chlorella* sp. cells and bacteria. In BR2, the presence of filamentous microalgae provided structural frameworks interconnecting the flocs, enhancing compactness, stability, and settling performance.

In TS2, by contrast, the presence of filamentous microalgae facilitated aggregation (as schematically illustrated in **Figure 13**) and decreased the abundance of free cells in effluents, raising recovery efficiency above 94% after settling and achieving complete removal by centrifugation. This corroborates previous findings that filamentous organisms act as structural frameworks for biomass aggregation (Wagner et al., 2015; Tiron et al., 2017). Moreover, in conventional activated sludge, filamentous bacteria are likewise regarded as the backbone of flocs, providing structural support for bacterial attachment and cohesion (Jenkins et al., 1993; Eikelboom and Geurkink, 2002). In addition, the predominance of filamentous microalgae in the TS2 effluent also contributed to the higher efficiency of the centrifugation step, as these forms were more easily separated from the liquid phase than the dispersed unicellular *Chlorella* cells in TS1.

Alongside the changes in phototrophic community composition, the protozoan community also exhibited a clear diversification in TS2. Microscopic investigations revealed free-swimming ciliates, crawling forms such as *Urostyla* sp., stalked ciliates (*Vorticella* sp. and *Epistylis* sp.), testate amoebae (*Arcella* sp.), and abundant rotifers, with eggs recorded in the later batches. This higher trophic complexity pointed to a more integrated and balanced ecological network compared to TS1, where protozoan diversity was limited. The presence of diverse protozoa is well recognized in traditional technology as primary bioindicators of the functional state of biomass and system performance (Madoni, 2011; Foissner, 2016). More recent findings also emphasize that protozoan predation can stimulate bacterial diversity and shape community structure in activated sludge, with positive effects on treatment performance (Burian et al., 2022). Taken together, these structural and trophic properties underline the capacity of the activated algae consortium to sustain a stable and functionally robust biomass, achieving performance levels comparable to a conventional activated sludge system while relying on an alternative biological system with distinct operational advantages such as the elimination of fossil fuel-based aeration requirements and the potential for biomass valorization.Overall, the study demonstrates that the tested optimized two-stage activated algae system achieved efficient COD and nutrient removal under nature-based oxygen supply, and improved microalgae biomass recovery under realistic operational conditions. By combining the functional contributions of bacteria and microalgae, and by leveraging community succession towards more cohesive phototrophic consortia, the process exhibited operational robustness and resilience without the need for mechanical aeration. While the study was conducted without parallel experimental replicates, the observed performance under the tested operating conditions supports the potential of microalgae-bacteria systems as sustainable alternatives to conventional activated sludge. Further research, including extended monitoring periods, will be essential to quantify variability and support advancement toward higher technology readiness levels, aligning wastewater treatment with the principles of circular bioeconomy and climate-resilient resource management.

## 5 Conclusions

This study evaluated the efficiency of using activated algae (microalgae–bacteria) biomass for raw dairy wastewater treatment, operated in a sequential two-stage configuration, to address the high organic load and elevated nutrient content of the influent, while relying exclusively on a natural source of oxygen sustained by photosynthesis. The process was proposed as an alternative to conventional biological treatment based on activated sludge. The system showed high removal efficiencies for organic matter and ammonium. Under the established operating conditions, aerobic processes were sustained by microalgae, highlighting the potential of this approach for nutrient removal without mechanical aeration. Beyond pollutant mitigation, the two-stage operation promoted enhanced microalgae biomass recovery, improved the efficiency of centrifugation, and reduced the hydraulic retention time by 50%. These findings emphasize the added value of integrating microalgae photosynthesis with bacterial activity in a functional consortium. Overall, the obtained performance represents an important step in advancing activated algae processes as viable alternatives to conventional biological treatment. By ensuring high treatment efficiency, facilitating biomass harvesting, and reducing operational demands, this approach supports the development of sustainable and resource-oriented strategies for nutrient-rich industrial effluents.

## Data Availability

The original contributions presented in the study are included in the article. Further inquiries can be directed to the corresponding author.
